# Novel Isosteviol-Based FXa Inhibitors: Molecular Modeling, In Silico Design and Docking Simulation

**DOI:** 10.3390/molecules28134977

**Published:** 2023-06-24

**Authors:** Marcin Gackowski, Burhanuddin Madriwala, Renata Studzińska, Marcin Koba

**Affiliations:** 1 Department of Toxicology and Bromatology, Faculty of Pharmacy, L. Rydygier Collegium Medicum in Bydgoszcz, Nicolaus Copernicus University in Torun, A. Jurasza 2 Street, 85089 Bydgoszcz, Poland; kobamar@cm.umk.pl; 2 Department of Pharmaceutical Chemistry, Faculty of Pharmacy, Nitte College of Pharmaceutical Sciences, Yelahanka, Bengaluru 560064, Karnataka, India; burhanmadri53@gmail.com; 3 Department of Organic Chemistry, Faculty of Pharmacy, L. Rydygier Collegium Medicum in Bydgoszcz, Nicolaus Copernicus University in Torun, A. Jurasza 2 Street, 85089 Bydgoszcz, Poland; rstud@cm.umk.pl

**Keywords:** thrombosis, FXa inhibitor, molecular modeling, isosteviol-like FXa inhibitors, docking simulation, artificial neural networks, QSAR

## Abstract

Direct oral anticoagulants are an important and relatively new class of synthetic anticoagulant drugs commonly used for the pharmacotherapy of thromboembolic disorders. However, they still have some limitations and serious side effects, which continuously encourage medicinal chemists to search for new active compounds acting as human-activated coagulation factor X (FXa) inhibitors. Isosteviol is a nontoxic hydrolysis product of naturally occurring stevioside and possesses a wide range of therapeutic properties, including anticoagulant activity. The present contribution describes the in silico design of novel oxime ether isosteviol derivatives as well as a molecular modeling approach based on QSAR analysis and a docking simulation for searching for novel isosteviol-based compounds as potential FXa inhibitors. The elaborated ANN model, encompassing topological and geometrical information, exhibited a significant correlation with FXa-inhibitory activity. Moreover, the docking simulation indicated six of the most promising isosteviol-like compounds for further investigation. Analysis showed that the most promising derivatives contain heterocyclic, aromatic, five-membered moieties, with substituents containing chlorine or fluorine atoms. It is anticipated that the findings reported in the present work may provide useful information for designing effective FXa inhibitors as anticoagulant agents.

## 1. Introduction

Direct oral anticoagulants (DOACs) are an important and a relatively new class of synthetic anticoagulant drugs commonly prescribed for the prevention of venous thrombosis following surgery or during atrial fibrillation, and for the treatment of venous thromboembolism (VTE) [1,2,3]. Dabigatran, released in 2010, is a direct thrombin inhibitor. While the DOACs that reversibly inhibit human-activated coagulation factor X (FXa) introduced to the pharmaceutical market after 2011 are rivaroxaban, apixaban, edoxaban, and betrixaban. FXa, in the complex with activated coagulation factor V, catalyzes the conversion of prothrombin to active thrombin, which in turn, is responsible for the transformation of fibrinogen into a fibrin insoluble in water, which results in the formation of a clot. DOACs exert their antithrombotic effect by inhibiting free and prothrombinase-bound FXa, thereby effectively blocking thrombus development and thrombin generation in a concentration-dependent manner. Activated factor Xa plays a key role in the blood-coagulation pathway as well as being an attractive target for anticoagulant drug development. However, its role is not restricted to the coagulation cascade, because it also stimulates multiple intracellular signaling pathways through G-protein-coupled protease-activated receptors (PARs) as well as inducing inflammatory and fibrotic responses [4]. At present, direct FXa inhibitors are among the most widely applied oral anticoagulants for the effective treatment of thromboembolic disorders. Interestingly, Eliquis^®^ (apixaban) was the fifth best-selling drug in the world in 2021 [5]. Moreover, xabans have been increasingly used as alternatives to vitamin K antagonists in the management of VTE and nonvalvular atrial fibrillation, and their use is considered the preferred anticoagulant therapy [3]. Historic anticoagulant use was accompanied with restrictions and inconveniences such as the risk of drug–food interactions (dietary influences) and the need for monitoring for potential dose adjustment. Through the administration of DOACs, characterized by a superior pharmacodynamic and predictable pharmacokinetic profile, these difficulties may be overcome. However, in the case of some of older patients, obese individuals, patients with renal impairment or requiring drugs that impact certain metabolic pathways, poor responders, or DOAC-treated individuals that require acute intervention, coagulation status should also be monitored. Thus, there has been an increasing demand for assessing the anticoagulant effects or levels of DOACs, either in emergency or routine clinical practice [6]. Despite significant advantages over traditional anticoagulants, there are some drawbacks and limitations of DOAC use, with bleeding complications being the main concern in clinical practice. Therefore, the efforts of medicinal chemists must be primarily directed towards the development of novel FXa inhibitors for the management of thromboembolic diseases.

**Figure 1:**
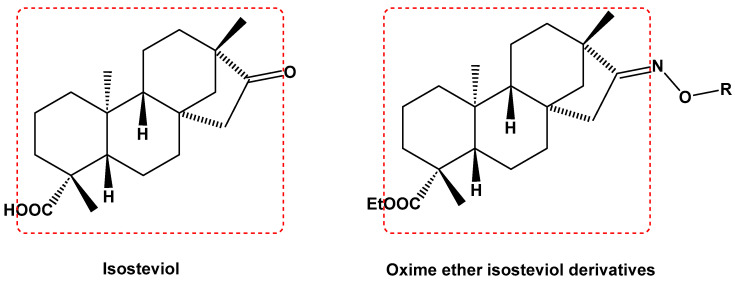
Structures of isosteviol and its oxime ethers derivatives.

In recent years, many efforts have been made to find novel, potent, and safer FXa inhibitors [7,8,9,10,11,12]. They include isosteviol (ISV) derivatives as natural-product-based compounds, which are a rich source of potential biological applications. Historically, natural products have contributed significantly to drug discovery and there is currently a renewed interest in natural products as drug leads [13]. Isosteviol (Figure 1) is an acid hydrolysis product of stevioside, which is a natural compound derived from the stevia plant with both hydrophobic and hydrophilic properties. Its skeleton may be used in the design and synthesis of other derivative compounds with potent bioactivities, which inspires and encourages medicinal chemists to design selective, potentially active, and polyfunctional isosteviol-like compounds for the management of different conditions. Moreover, thanks to its relatively low toxicity, low cost, rigidity, and chiral framework, it has been recognized for its extensive bioactivity. Therefore, isosteviol has proved its usefulness and justified its position in the field of medicinal chemical research [14,15].

The development of new drugs is a complex, time-consuming, tedious, and cost-intensive process. This process costs millions of dollars and man-hours, and still, the drug might not work [16]. And that is why there is an enormous need to use molecular modeling methods to facilitate the discovery process of new drugs. Molecular modeling methods are of great importance in research as well as an important and essential tool for medicinal chemists in the drug design process. There are numerous possible methods based on theoretical chemistry and applications of molecular modeling [17,18]. Some of them, such as structure and visualization, conformation analysis, the prediction of pharmacological activities, and molecular interactions are utilized and described in the present work. Molecular modeling enables the generation and optimization of three-dimensional structures of compounds and the prediction of molecular and biological properties. Moreover, molecular docking is the most common approach to structure-based drug design due to the ability to predict the binding-conformation of small-molecule ligands to the appropriate target binding site.

Since isosteviol-like compounds are considered to be promising antithrombic agents, in the present work, a molecular modeling approach based on the establishment of a model reflecting quantitative structure—activity relationships was implemented to predict anticoagulant activity of novel isosteviol compounds. A docking simulation was carried out to better understand the binding mechanism and to identify the key features enhancing the ligand–receptor binding affinity. Moreover, novel oxime ether isosteviol derivatives (Figure 1) were designed in silico using computational and empirical medicinal chemistry strategies.

## 2. Results

### 2.1. Molecular Modeling

**Figure 2:**
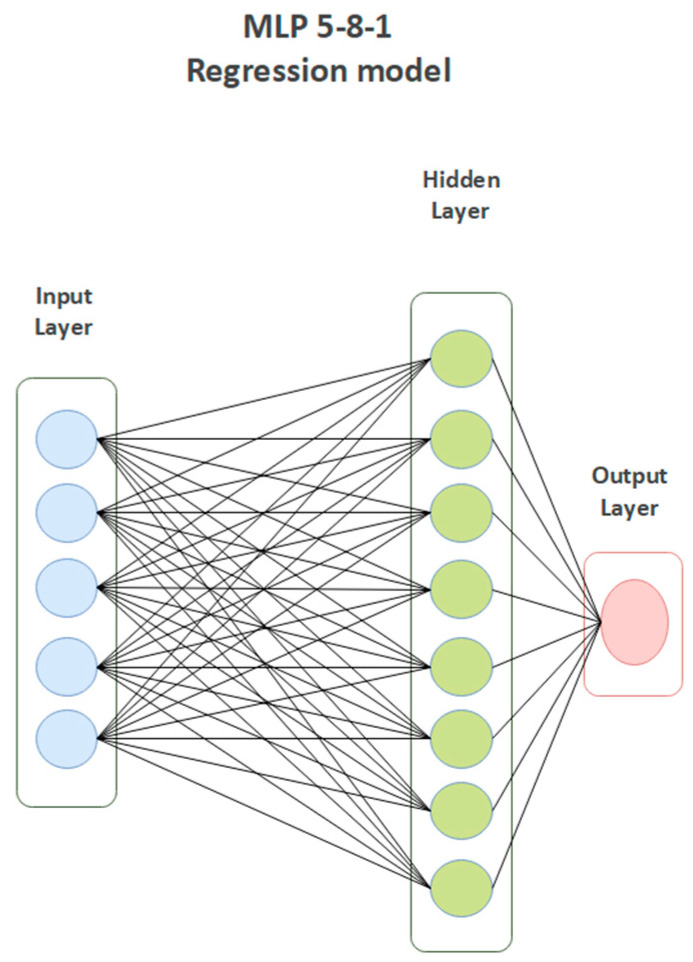
Graphical representation of regression MLP 5-8-1 model.

The application of an automated network search algorithm enabled the training of about one hundred of neural networks characterized with different qualities. On the basis of training, test and validation performance was denoted as correlation coefficient as well as training, test, and validation sum of squares error. A multilayer perceptron (MLP) 5-8-1 network that best reflects relationships between preselected descriptors and FXa-inhibitory activity was selected to solve the studied regression problem. Many different algorithms and activation functions were used in the course of network creation. For the predictive network, a Broyden–Fletcher–Goldfarb–Shanno (BGFS) 31 learning algorithm, a hyperbolic tangent function, and logistic activation functions were used. The network is composed of five artificial neurons forming the input layer, eight artificial neurons that make up the hidden layer, and one neuron acting as the output layer (Table 1). The complexity of the network architecture is presented in Figure 2.

**Figure 3:**
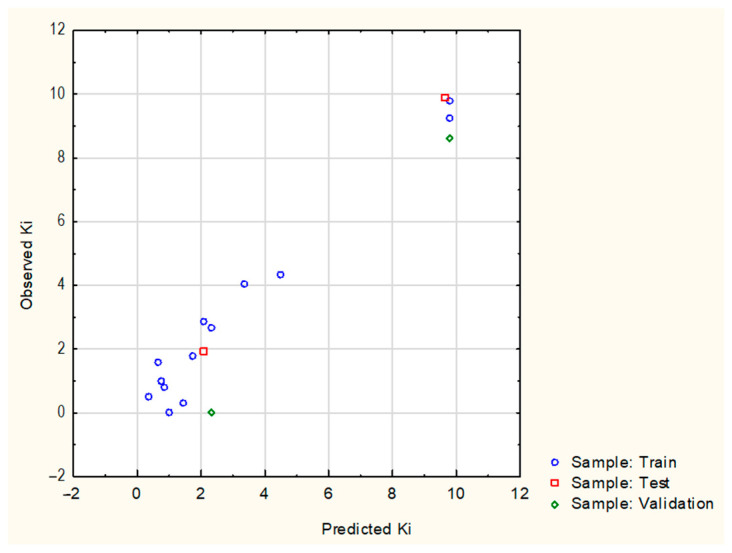
Graphical representation of regression analysis: training set R = 0.981058; test set R = 0.999999 and validation set R = 0.999999.

This predictive ANN MLP 5-8-1 model is notable for a high correlation between the actual K_i_ value and the value predicted by the network of 0.981058 for training, 0.999999 for the test set, and 0.999999 for validation. The values of the sum of squares error expressing the difference between the observed values and those predicted by the elaborated regression model were 0.181916 for the training set, 0.022793 for the test set, and 1.66782 for the validation. Graphical representation as a plot of predicted values of K_i_ versus actual values of K_i_ confirms that the network predicts the series data reasonably well. The results of the correlation between the experimentally determined K_i_ values and those predicted by the MLP 5-8-1 network model are graphically presented in Figure 3.

As a consequence of variable preselection for further model building, five molecular descriptors were introduced as input variables. Three out of the five preselected descriptors belonged to 3D geometrical descriptors and the remaining two were 2D topological descriptors. This indicates that two- and three-dimensional molecular structures are the most important for the anticoagulant activity of studied ISV compounds. They are representatives of GETAWAY descriptors, 2D autocorrelations, and 2D matrix-based descriptors. ANN regression analysis and consequent sensitivity analysis revealed the importance of particular descriptors for the FXa-inhibitory activity, on the basis of the highest error quotient. The sensitivity analysis helped in determining the molecular features which have the strongest effect the anticoagulant activity of isosteviol compounds. The rank list of descriptors incorporated into the MLP 5-8-1 model, in descending order of importance along with their dimensionality, block, and definition is presented in Table 2. An error quotient for each variable entering the model exceeded value 1, which means that all preselected descriptors were significant for the MLP 5-8-1 regression model. Otherwise, a given variable should be excluded due to no effect or a deterioration of the performance of the network.

Chen et al. reported that 19-ethyl ester and 16-oxime groups are the key to anticoagulant activity [10]. Moreover, it has been observed that oxime ether derivatives possessing a thiophene group or a 3-methyloxetane group exhibit excellent in vitro activity against human FXa. On the basis of the observations mentioned above and structure–activity relationships established in the present investigation, 26 new oxime ether isosteviol derivatives were designed, containing moieties with saturated and unsaturated alicyclic and heterocyclic four- and five-membered rings with sulphur, oxygen, or nitrogen atoms (Table 3).

Predicting the of activity of novel oxime isosteviol analogues e1–e26 against human-activated FXa led to the attainment of interesting results. According to the K_i_ values presented in Table 3 some newly designed compounds may be potential inhibitors of FXa.

### 2.2. Molecular Docking

Molecular docking was used to examine the molecular interaction and affinity of the examined isosteviol compounds and conventional medicines for binding to human-activated coagulation factor X protein, and to prioritize the designed compounds. The set of compounds showed scores close to standards, as indicated in Table 4. Compounds e15 and e21 had the lowest binding-free energy (delta G) of all the compounds in the set, at −8.3 kcal/mol, which is close to the standard of edoxaban. However, their binding affinity was slightly weaker than apixaban and rivaroxaban. Similarly, compounds e20, e23, e24, and e25 also showed scores close to those of e15 and e21. For each reduced complex, the interaction energy consisted of van der Waals, electrostatic, and intermolecular hydrogen-bonding energies. For ligand-binding inside the active site via, for instance, hydrophobic interactions and hydrogen bond interactions in practically all complexes, the predicted residues were energetically significant.

**Figure 4:**
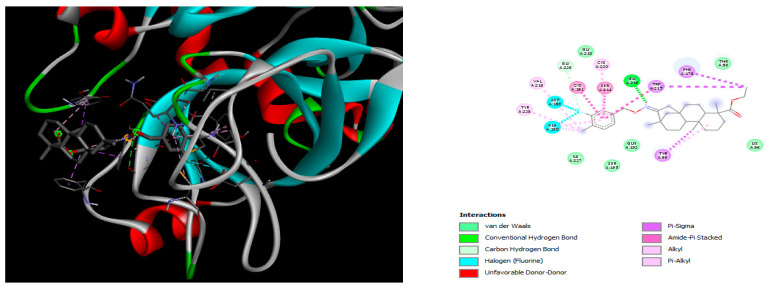
The 3D and 2D interactions of compound d with human-activated coagulation factor X protein.

**Figure 5:**
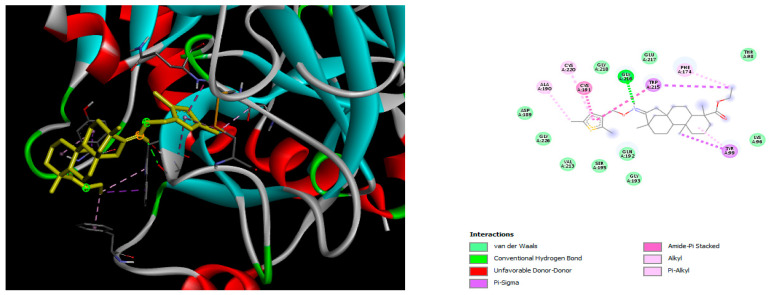
The 3D and 2D interactions of compound e15 with human-activated coagulation factor X protein.

**Figure 6:**
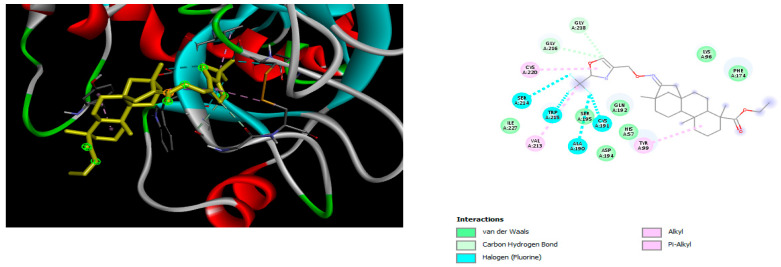
The 3D and 2D interactions of compound e21 with human-activated coagulation factor X protein.

**Figure 7:**
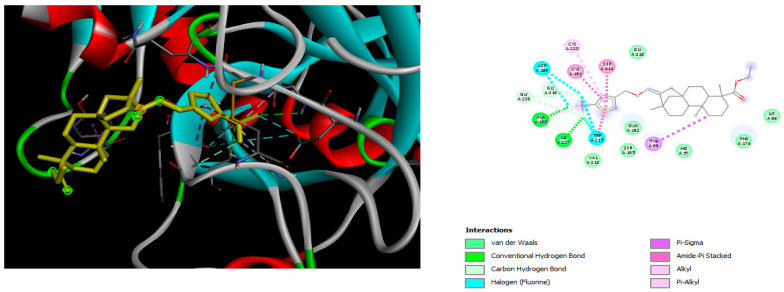
The 3D and 2D interactions of compound e23 with human-activated coagulation factor X protein.

**Figure 8:**
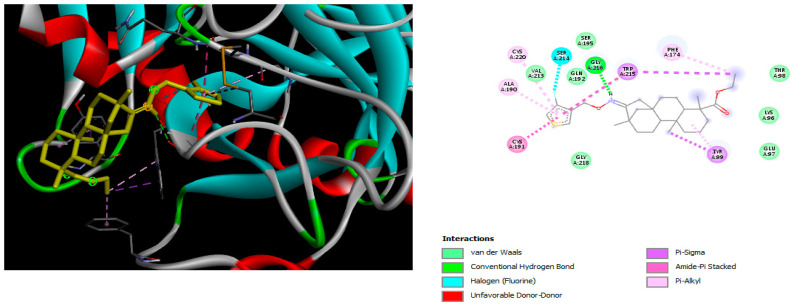
The 3D and 2D interactions of compound e24 with human-activated coagulation factor X protein.

**Figure 9:**
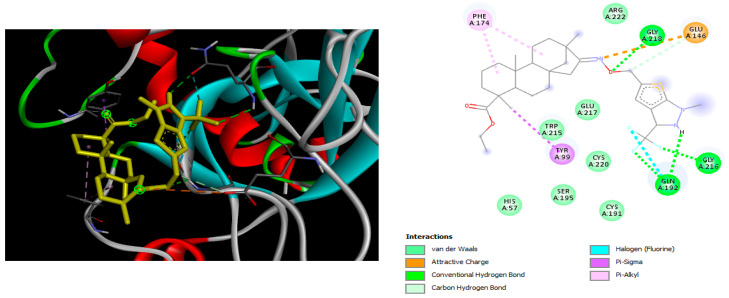
The 3D and 2D interactions of e25 with human-activated coagulation factor X protein.

**Figure 10:**
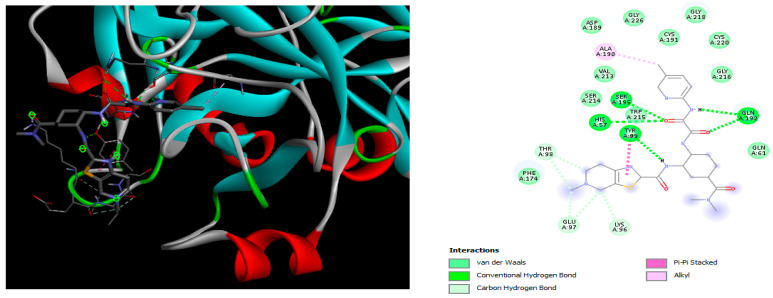
The 3D and 2D interactions of edoxaban with human-activated coagulation factor X protein.

The docking simulation reveals that all isosteviol derivatives in Figure 4, Figure 5, Figure 6, Figure 7, Figure 8, Figure 9 and Figure 10 show similar interaction with FXa protein, in which the fused polycyclic region is involved in molecular interaction with S4 pocket and derivative part is involved in the S1 and the S2 site of the protein.

The binding model of compound e15 exposed unique Pi-sigma interaction between tryptophan 215 and pyrrole moiety as well as the ester group in the S1 region. Tyrosine 99 also showed Pi-sigma bond with the alkyl group of the polycyclic ring (Figure 5).

A trifluoro interaction of the isoxazole derivative was observed with amino acids, Ser214, Trp215, Cys191, and Ala190 in the S1 and the S2 region of the protein (Figure 6)

In the case of compound e23, the amino acids, Asp189 and Trp215, showed possible H-bonds with the trifluoro derivative of the pyrrole ring (Figure 7).

The fluorine atom of the furan ring of compound e24 showed interaction with serine 214 in the S2 site, possibly due to an H-bond between fluorine and the thiol group of serine (Figure 8).

The fluorine atom of compound e25 showed bonding with glutamine, possibly due to an H–Bond between fluorine and carbonyl oxygen of glutamine (Figure 9).

In the case of edoxaban, there was conventional H-Bonding in the S4 region of the protein between the thiophene ring and Tyr99. Two carbonyl groups of the ligand were involved in the conventional H-bond and the van der Waals interaction in the S1 and S2 regions with Ser195, Gln192, and His57 (Figure 10).

The docked structures depicted in Figure 4, Figure 5, Figure 6, Figure 7, Figure 8 and Figure 9 show negative binding-free energies which indicates the possibility of stable interactions between the ligand and the protein target. Moreover, additional interactions were also found in comparison to edoxaban (standard drug), so the complexes’ stability results can also be confirmed with extra Halogen, Attractive Charge, Pi-Sigma, Pi-Alkyl, unfavorable donor–donor, and amide–pi stacked interactions.

## 3. Discussion

An isosteviol core is considered to be important and effective in the area of medicinal chemical research and numerous ISV-like compounds have been synthetized through its chemical modification [15]. Synthetic and semisynthetic ISV derivatives exhibit numerous therapeutic properties including anticoagulant activity defined as FXa inhibition, as described in the present work.

Application of artificial neural networks have led to the establishment of the significance model for solving regression problems and reflecting quantitative structure–activity relationships of studied isosteviol-like compounds. The ANN model incorporates five descriptors selected from a considerable descriptor matrix (Table 2), with the aims of avoiding overtraining and indicating the most important molecular properties affecting the FXa inhibitory activity of ISV compounds. According to the preselection process, descriptors pertaining to the polarizability, ionization potential, and van der Waals volume of the compounds influenced the inhibitory activity against human-activated blood coagulation factor X. These indices encode 3D and 2D information from the molecular structure, which seems to provide a model with acceptable predictivity, because the modelled activity not only depends on the 3D features of the molecule. Three of the five descriptors contributing to the QSAR model are the three dimensional GETAWAY (GEometry, Topology, and Atom-Weights AssemblY) descriptors matching geometrical information derived from the influence matrix, topological information derived from the molecular graph, and chemical information from selected atomic properties [20,21,22]. H-GETAWAY indices are obtained from a new representation of molecular structure, the molecular influence matrix, through the values of atomic Cartesian coordinates. While R-GETAWAY descriptors are calculated from the influence/distance matrix R, where the elements of the molecular influence matrix are combined with those of the geometry matrix. Moreover, they cover local or distributed information on molecular structure, so it is beneficial when more than one GETAWAY index is present in a QSAR model, as in the case of the predictive ANN model. HATS2i and HATS2e descriptors are calculated on the basis of the leverage weighted autocorrelation of molecular graphs, and as atomic weights, ionization potential, and Sanderson electronegativity are used for their calculation, respectively. While R5e denotes R autocorrelation of lag 5 weighted by Sanderson electronegativity and shows the importance of electronic properties of compounds when interacting with the molecular target. Thetwo remaining descriptors (GATS8p and SpMAD_B(v)) refer to a 2D representation of a molecule. The GATS8p descriptor was the most influential variable in the ANN model, as it occupies the first place in the rank list (Table 2). It belongs to the 2D-autocorrelation descriptors, which describe how the considered property is distributed along the topological structure. GATS8p associates the presence of polarizable pairs of atoms, at a specific topological distance, with the pharmacological activity of compounds [23]. It indicates the presence of polarizable pairs of atoms eight bonds apart. The SpMAD_B(v) descriptor is the last on the rank list, but is still significant for the ANN model. It represents the spectral mean absolute deviation from the Burden matrix, weighted by van der Waals volume. This index belongs to the 2D-matrix-based descriptors, which convey the information about the molecular branching and cyclicity of the molecules [24]. They are obtained by applying a set of basic algebraic operators to different graph-theoretical matrices representing an H-depleted molecular graph of molecules [25].

The length and expense of the drug development process are reduced with the frequent and widespread application of virtual screening approaches [26]. Finding novel ligands for biological proteins is done using the molecular docking method, which is crucial for both structure- and ligand-based drug design [27]. To better understand the chemical underpinnings of the biological activity of isosteviol derivatives, the approach has been effectively applied to the theoretical prediction of ligand–target interactions. The probable mode of action and mode of binding of active compounds to the FXa protein have also been clarified. Some active molecules were discovered in the set of 26 newly designed isosteviol-like compounds when were docked, using the PyRx tool. These molecules produced better molecular interactions and lower binding energies (from −8.3 to −6.9 kcal/mol) with the selected protein. With a lowering of a docking score, the isosteviol compounds’ affinity for the FXa receptor rises, resulting in more effective bioactive chemicals.

The abovementioned structure–activity considerations mainly refer to electronic properties, branching and cyclicity, the presence of polarizable pairs of atoms, ionization energy, and electronegativity. Chen et al. synthesized several ISV-like compounds and observed that the 19-ethyl ester group and the 16-oxime group present in the isosteviol core are essential for the anticoagulant activity [10]. Moreover, a thiophene, a 3-methyloxetane, or a pentafluorobenzene group can enhance this activity, whereas Shi et al. reported that a phenyl group containing an electron-donating group reduced the inhibitory activity; quite the reverse—a phenyl group containing a chlorine atom, in the case of thiourea isosteviol derivatives, led to the enhancement of the FXa-inhibitory activity [28]. The designed ANN model gave an insight into the potential FXa-inhibitory activity of the designed ISV compounds, emphasizing the role of the branching of a molecule as well as electronegative and polarizable atoms. Moreover, docking simulation was conducted to identify the key features required to design novel candidates and to explore the interaction mechanism between the compounds and FXa protein. According to the docking simulation carried out for ISV compounds with experimentally tested FXa-inhibitory activity, it was observed that halogen, especially fluorine, may enhance the ligand–receptor binding affinity. This phenomenon was confirmed in the case of the newly designed ISV compounds containing at least one atom of halogen, which exhibited the lowest docking scores (e15, e20, e21, e23, e24, and e25). For instance, compound e15, with a thiophene ring substituted with two chlorine atoms, had a slightly lower docking score than compound k, possessing an unsubstituted thiophene ring. Similarly, compound e24 showed a slightly better binding affinity. Additionally, graphical representations of molecular interactions (Figure 4, Figure 5, Figure 6, Figure 7, Figure 8, Figure 9 and Figure 10) confirm that the presence of an electronegative halogen atom resulted in interactions such as halogen (fluorine) and conventional hydrogen bonds that may be responsible for better ligand–receptor binding affinities. In view of the above, six ISV-like candidates for synthesis and further investigations with promising anticoagulant activity have been indicated on the basis of the conducted docking simulation (e15, e20, e21, e23–e25).

## 4. Materials and Methods

### 4.1. Molecular Modeling

#### 4.1.1. Energy Optimization of Isosteviol Analogues

HyperChem 8.0 (Hypercube Inc., Gainesville, FL, USA) software was used for visualization of studied molecules and calculations of the minimum energy state. The studied set included seventeen isosteviol ((4α,8β,13β)-13--Methyl-16-oxo-17-norkauran-18-oic acid) derivatives, which exhibited in vitro activity against human FXa, denoted as inhibition constant K_i_, as shown by Chen et al. [10] (Table 5). In the first step, optimization was accomplished by applying the molecular mechanics method using MM+. Subsequently, the investigated structures in ground state were optimized using Austin Model 1 (AM1) method with restricted Hartree–Fock (RHF) basis. Both optimizations were obtained by applying the Polak–Ribiere (conjugate gradient) method, and the RMS gradient was set to 0.01 kcal/(Å mol) or 30,000 maximum cycles.

**Table 5:** Chemical structures and biological activity of studied compounds.

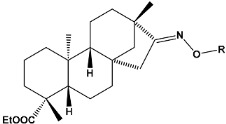				
Compound	Sample	R *	Optimized Molecular Structure	Inhibition Constant (K_i_), µM **
a	train	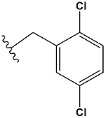	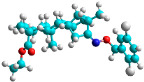	9.253
b	train	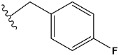	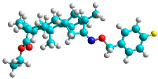	4.333
d	train	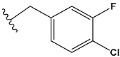	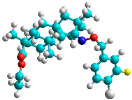	9.786
e	train	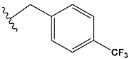	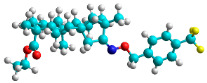	2.693
f	train	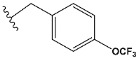	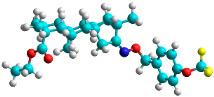	1.023
g	train	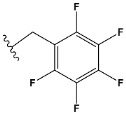	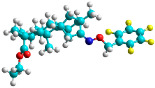	0.321
h	test	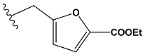	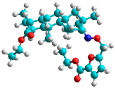	9.877
i	train	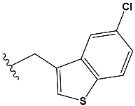	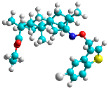	0.515
j	test	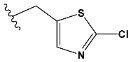	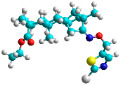	1.941
k	train	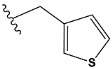	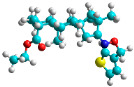	0.015
l	train	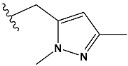	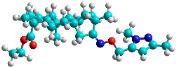	4.025
m	train	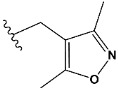	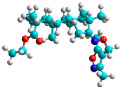	2.875
n	train	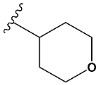	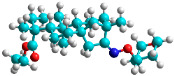	1.809
o	train	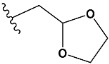	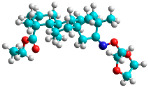	1.612
p	validation	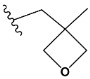	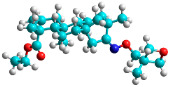	0.028
q	train	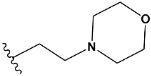	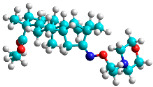	0.785
r	validation	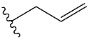	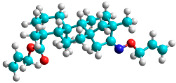	8.607

* a side chain of a particular oxime ether isosteviol analogue; ** molarity.

#### 4.1.2. Molecular Descriptors

In order to obtain mathematical representations of a molecule’s chemical information, 4885 molecular descriptors were calculated for studied compounds at minimum energy state with the use of Dragon 7 (Talete, Milano, Italy) software. These descriptors represent 29 logical blocks and belong to different classes: 0D constitutional, 1D structural, 2D topological, and 3D geometrical descriptors [19]. In fact, some of 4885 calculated descriptors were removed from the descriptor matrix undergoing regression analysis in order to assure high model performance. Reasons and numbers of descriptors removed and saved for further analysis are presented in Table 6.

#### 4.1.3. Regression Analysis

**Figure 11:**
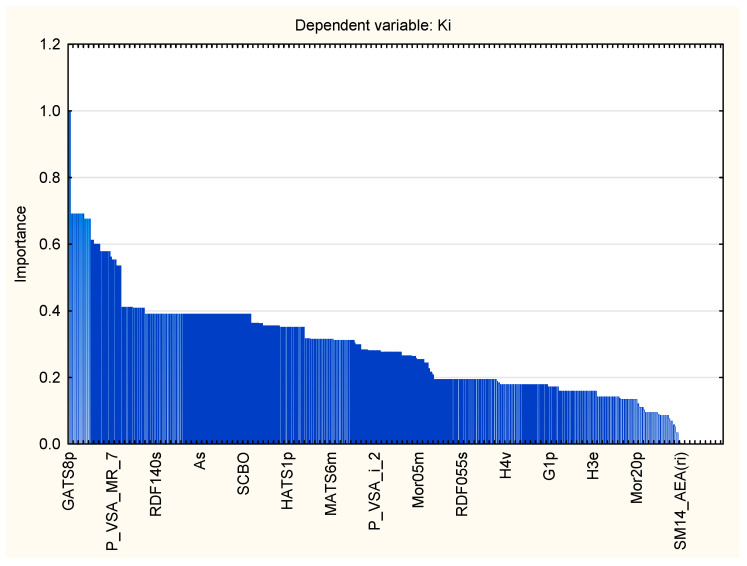
Descriptor importance plot.

Statistica 13.3 software (TIBCO Software Inc., Palo Alto, CA, USA) was used for the purpose of statistical analysis. In particular, algorithms implemented in the Statistica Random Forest were applied for the preselection of 5 the most important descriptors from 1274 candidates in order to decrease complexity of the network and increase its predictive performance (Figure 11 and Table 7). It should be emphasized that most representative molecular features in relation to the examined activity should be used as input in the building of an ANN [29].

Random Sample Selection module in Statistica 13.3 Data Miner was used to divide the whole set of isosteviol derivatives into a training (for network learning), a test (for the final cross-evaluation of network quality), and a validation group (to control the effects of learning algorithm). In the next step, further post-processing algorithms, i.e., STATISTICA automated neural networks (SANN), were applied to establish relationships between molecular descriptors representing molecules’ properties and FXa-inhibitory activity. The elaborated model solves regression problem, so the response of the network is qualitative, denoted as the inhibition constant.

During the network elaboration, the molecular descriptors are placed in the input units, and then the hidden and output layer units are progressively executed in their sequential order. Each of them computes its activation value by taking the weighted sum of the outputs of the units in the preceding layer. The activation value is passed through the activation function to produce the output of the neuron. When the entire network has been executed, the neurons of the output layer are assigned to a desired response (FXa-inhibitory activity) of the entire network [29,30]. Automated network search (ANS) scaled the input and target variables, and enabled the creation of a variety networks differing in activation and error functions, or network complexity. Parameters such as network size and training algorithm control parameters must be user-defined. SANN supports most important classes of neural networks such as multilayer perceptrons, radial basis function networks, and Kohonen self-organizing feature maps. After network training, the best active networks were displayed to choose the network with the best performance, avoiding a lengthy trial-and-error process [30]. In addition, the sensitivity analysis was conducted to assess whether the variables were significant for the predictive ANN model. Finally, the designed model was applied for the purpose of predicting the FXa activity of newly designed isosteviol derivatives (e1–e26).

### 4.2. Molecular Docking Study

**Figure 12:**
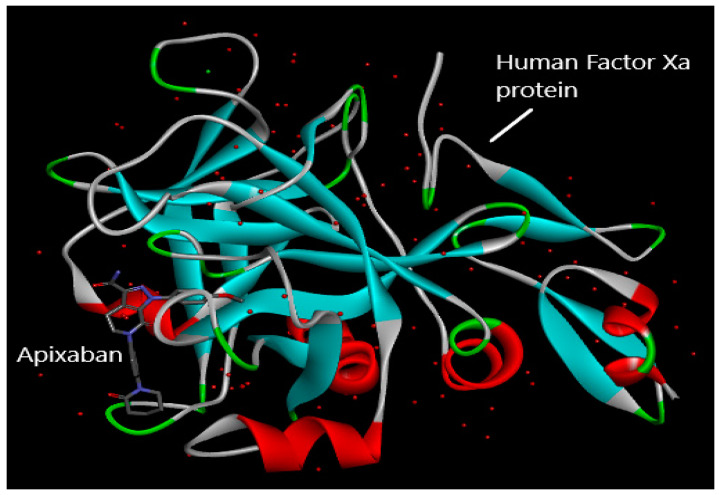
Structure of human-activated coagulation factor X protein in complex with apixaban inhibitor taken from RCSB Protein Data Bank (PDB ID: 2P16).

A sophisticated computer approach called molecular docking was used to examine the interactions between therapeutically useful compounds or medicines and the proteins implicated in disease pathogenesis. The medication is referred to as a ligand because it interacts with the protein’s binding or active site in very stable conformations, using the least energy. These potential drug-binding conformations are referred to as docking poses, and each one has a unique energy level known as its binding-free energy or docking score, that is stated in terms of kcal/mol unit. In terms of ligand–protein interaction, the conformation of the molecule with the lowest docking score is thought to be the most efficient and stable. This conformation can then be employed in pharmacophore modeling or QSAR studies to optimize the intended therapeutic compounds [31]. In this study, human activated coagulation factor X protein complexed with apixaban inhibitor was selected (PDB ID: 2P16), and its X-Ray-diffraction structure was taken from the RCSB Protein Data Bank in PDB format (Figure 12) [32].

**Figure 13:**
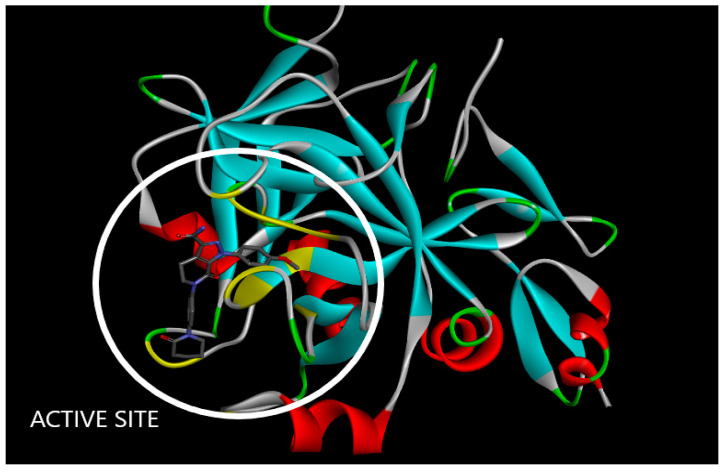
The structure of protein showing the active or binding site of human-activated coagulation factor X protein for the molecules to interact.

The AutoDock Vina programme was used to prepare the protein (The Center for Computational Structural Biology, La Jolla, CA, USA). This involved the removal of water molecules, undesirable residues, identical amino acid chains, another inhibitor (apixaban) already present in the protein, as well as the assignment of Kollmann and Gasteiger charges. After that, the protein was stored in PDB file format [33]. Figure 13 presents the protein structure illustrating the human-activated coagulation factor X protein’s active or binding location for the molecules to interact.

For docking simulation, a total of 42 ligands (ISV compounds including 26 newly designed compounds with the most promising FXa inhibitory activity predicted by the elaborated ANN model) were prepared. The results were compared with the standard drugs, apixaban, edoxaban, and rivaroxaban, and were saved using BIOVIA Discovery Studio 2021 Client (Discngine S.A.S., Paris, France). The structures of the standard pharmaceuticals were retrieved from Pubchem and saved in 3D conformer as SDF format (later converted to PDB format) [33]. The standard drugs were chosen for comparison of docking scores. Using Discovery Studio 2021 Client, the binding site of the protein was determined based on the location of the apixaban inhibitor that was already present. PyRx software was loaded with the protein’s structure and two sets of ligands separately (SourceForge, San Diego, CA, USA). The ligands were energy-reduced before being transferred to PDBQT file format along with the protein. According to the dimensions of the protein’s active site, as determined by Discovery Studio 2021 Client, the grid box was placed in the protein to identify the binding spot for the ligands to interact or dock [34]. The grid box’s dimensions were 25 × 25 × 25, and its centre was located at X = 9.54, Y = 43.27, and Z = 63.47. After setting grid size, the Vina programme in PyRx was used to simulate molecular docking. All of the ligands were docked when the process was allowed to run, and each one produced nine conformations and matching docking scores. Using the Discovery Studio 2021 Client, the docking interactions of every molecule with the protein were shown in 2D and 3D images.

## 5. Conclusions

QSAR and molecular docking techniques were successfully applied on oxime ether isosteviol derivatives as potent anticoagulant agents, in order to produce a model that relates the chemical structures and FXa inhibitory activities of examined molecules. A predictable model was generated using an artificial neural network algorithm with Statistica software. The model revealed that the FXa-inhibitory activity of isosteviol compounds is influenced by following descriptors appearing in descending order: GATS8p, HATS2i, R5e, HATS2e, and SpMAD_B(v). The docking simulation was applied in order to better understand the binding mechanism and to identify the key features enhancing the ligand–receptor binding affinity. The combined use of both techniques facilitated the in silico design of novel isosteviol-like compounds. Based on the results, the FXa activity of proposed molecules was predicated by the elaborated QSAR model. Moreover, docking analysis indicated six compounds as possible lead molecules, which can be further optimized for an effective pharmacodynamic and pharmacokinetic profile to produce the potent anticoagulant activity. The conducted research indicates that the most promising FXa inhibitors are isosteviol derivatives, containing thiophene, furan, and oxazole rings, or condensed thiophene and pyrazole moiety with the presence of the electron-withdrawing group, e.g., chlorine, fluorine atoms, or trifluoromethyl substituents. These findings may be helpful in early anticoagulant drug development.

## Figures and Tables

**Table 1:** Learning algorithm and activation functions used for network development.

MLP 5-8-1 Model	
Learning algorithm	BFGS 31
Activation function (Hidden layer)	Logistic
Activation function (Output layer)	Tanh

**Table 2:** Sensitivity analysis results for MLP 5-8-1 model.

Name	Definition	Category	Dimensionality	Error MLP 5-8-1	Rank
GATS8p	Geary autocorrelation of lag 8 weighted by polarizability [19]	2D autocorrelations	2D	16.75531	1
HATS2i	leverage-weighted autocorrelation of lag 2/weighted by ionization potential [19]	GETAWAY descriptors	3D	14.49011	2
R5e	R autocorrelation of lag 5/weighted by Sanderson electronegativity [19]	GETAWAY descriptors	3D	13.33332	3
HATS2e	leverage-weighted autocorrelation of lag 2/weighted by Sanderson electronegativity [19]	GETAWAY descriptors	3D	8.866242	4
SpMAD_B(v)	spectral mean absolute deviation from Burden matrix weighted by van der Waals volume [19]	2D-matrix-based descriptors	2D	8.699860	5

**Table 3:** Designed isosteviol structures bearing oxime ether moiety with FXa inhibitory activity predicted by the MLP 5-8-1 model.

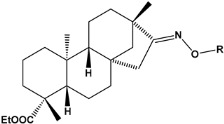		
Compound	R *	Predicted Inhibition Activity Against FXa: Inhibition Constant (K_i_), [µM] **
e1	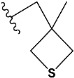	9.785990
e2	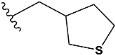	1.118201
e3	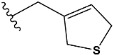	0.645249
e4	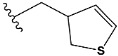	9.282278
e5	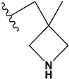	3.984843
e6	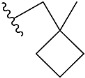	6.454281
e7	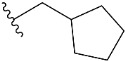	2.640193
e8	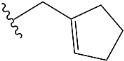	1.421969
e9	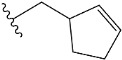	4.412301
e10	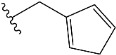	1.569841
e11	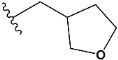	1.133094
e12	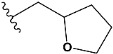	0.936761
e13	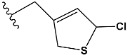	0.372101
e14	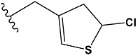	0.544725
e15	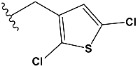	0.906731
e16	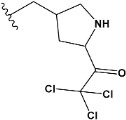	0.947983
e17	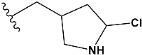	0.936441
e18	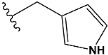	0.650819
e19	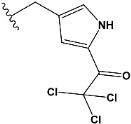	0.493827
e20	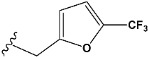	0.673789
e21	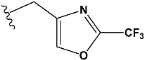	0.656182
e22	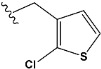	0.749590
e23	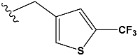	0.554588
e24	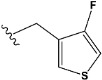	0.642575
e25	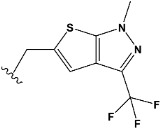	0.687738
e26	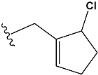	0.854700

* a side chain of a particular oxime ether isosteviol analogue; ** molarity.

**Table 4:** Docking scores of set of isosteviol derivatives and standard drugs against FXa protein (newly designed compounds with the highest affinity for the FXa receptor are marked in yellow).

Compound	Binding-Free Energy (kcal/mol)
a	−8.7
b	−8.3
d	−9.3
e	−8.1
f	−8.8
g	−8.3
h	−8.4
i	−7.4
j	−8.0
k	−8.1
l	−8.7
m	−7.8
n	−6.9
o	−7.7
p	−7.0
q	−8.0
r	−6.8
e1	−6.9
e2	−7.7
e3	−8.0
e4	−7.4
e5	−7.2
e6	−7.1
e7	−7.4
e8	−7.4
e9	−7.5
e10	−7.6
e11	−7.3
e12	−7.7
e13	−7.7
e14	−7.2
e15	−8.3
e16	−6.9
e17	−7.7
e18	−7.4
e19	−6.9
e20	−8.1
e21	−8.3
e22	−7.0
e23	−8.1
e24	−8.2
e25	−8.2
e26	−7.0
apixaban	−10.3
edoxaban	−8.8
rivaroxaban	−9.4

For the structures of a–r compounds see Table 5, Materials and Methods section.

**Table 6:** Number of descriptors included and excluded from statistical analysis.

Action	Reason	Number
deleted	constant	1856
	near constant	95
	all missing	2
	one missing	2
	highly correlated (|r| > 0.95)	1658
	standard deviation < 0.0001	1856
retained	suitable for model-building	1274

**Table 7:** Molecular descriptors used to design ANN models.

Symbol	Variable Rank	Importance
GATS8p	100	1
R5e	100	1
HATS2i	100	1
HATS2e	100	1
SpMAD_B(v)	100	1

## Data Availability

The data presented in this study are available on request from the corresponding author.
